# High Ghrelin Level Predicts the Curve Progression of Adolescent Idiopathic Scoliosis Girls

**DOI:** 10.1155/2018/9784083

**Published:** 2018-07-16

**Authors:** Hong-Gui Yu, Hong-Qi Zhang, Zhen-Hai Zhou, Yun-Jia Wang

**Affiliations:** Department of Spine Surgery, Xiangya Hospital, Central South University, China

## Abstract

**Background:**

Adolescent idiopathic scoliosis (AIS) is common deformity with unknown cause. Previous studies have suggested the abnormal serum leptin and ghrelin level in AIS girls. The aim of present study was to evaluate whether the serum leptin and ghrelin level could serve as risk factor in predicting the curve progression in AIS girls. The associations between them and the physical characteristics were also investigated.

**Materials and Methods:**

Circulating leptin and ghrelin levels from 105 AIS girls and 40 age-matched non-AIS girls were examined by enzyme-linked immunosorbent assay. The correlations between ghrelin and leptin levels and growth-related parameters (age, weight, corrected height, corrected BMI, main Cobb angle, and Risser sign) were analyzed in AIS group. Multivariate logistic regression was used to investigate factors predicting curve progression in AIS girls.

**Results:**

A significantly lower leptin level (6.55 ± 2.88 vs. 8.01 ± 3.12 ng/ml,* p* < 0.05) and a higher ghrelin level (6.33 ± 2.46 vs. 4.46 ± 2.02 ng/ml,* p* < 0.05) were found in all AIS patients, as compared with normal controls. Curve progression patients had a higher ghrelin level than stable curve patients (7.61 ± 2.48 vs. 5.54 ± 2.11 ng/ml,* p* < 0.01); for leptin level, there was no significant difference between progression and stable group. The results of multivariate logistic stepwise regression showed that premenarche status, initial main Cobb magnitude that was more than or equal to 23°, high ghrelin level (≥7.30 ng/ml), and lower Risser grade (grades 0 to 2) were identified as risk factors in predicting curve progression. Ghrelin levels of >6.48 ng/ml were predictive for curve progression with 70.00 % sensitivity and 72.31 % specificity, and the area under the curve (AUC) was 0.741 (95 % confidence interval 0.646-0.821).

**Conclusions:**

High ghrelin level may serve as a new quantitative indicator for predicting curve progression in AIS girls.

## 1. Introduction

Adolescent idiopathic scoliosis (AIS) is a complex 3-dimensional spinal deformity occurring predominantly in girls during peripubertal age with unknown cause [1]. Epidemiologic studies showed that the prevalence of AIS is 1.5% to 3.0%, and 10% to 15% of AIS patients demonstrate curve progression [2, 3]. Progressive scoliosis may result in severe deformity, back pain, cardiac dysfunction, and pulmonary constraint [4]. Among 14- to 15-year-old girls, the prevalence is up to 13.81% [5]. Hence, it is important to predict which curves will progress so that appropriate and timely treatment can be provided.

Several clinical and genetic parameters for predicting curve progression have been proposed like curve-related factors, maturity-related factors, osteopenia, SNPs, and others [6–8]. Unfortunately, the ability of the individual parameter is not accurate enough in predicting curve progression in AIS patients because of substantial variability [9]. In a review to evaluate published data on the prediction of progressive AIS, it concluded that presented reported predictors have low level of evidence due to the deficiency of observational studies [10]. The ScoliScore test developed by Axial Biotechnology is only effective in predicting the outcome of white AIS patient [11]. Therefore, it is still necessary to explore new effective risk factors for progressive AIS patients.

Leptin is a 16-kDa peptide hormone secreted principally by the white adipose tissue [12]. It displays an appetite-suppressing effect which can reduce the formulation and accumulation of fat. Ghrelin, a 28-amino acid predominantly produced by the gastric mucosa [13], has been shown to display appetite-stimulatory effects and induces a positive energy balance leading to body weight gain. Leptin and ghrelin antagonize each other in regulating energy balance [14]. A number of studies have reported serum leptin and ghrelin level disorders in AIS patients [15–20]. However, the significance of leptin or ghrelin as a biomarker in progressive AIS patients has not yet been reported.

The aim of the present study was to evaluate whether the serum leptin or ghrelin level could serve as risk factor in predicting the curve progression in AIS girls. The associations between them and the physical characteristics were also investigated.

## 2. Materials and Methods

### 2.1. Ethics Statement

Ethics approval for the present study was obtained from the Ethics Committee of Xiangya Hospital of Central South University (Changsha, China). Procedures involving human subjects were performed in accordance with the Code of Ethics of the World Medical Association (Declaration of Helsinki). Written informed consent was obtained from all the subjects as well as from their legal guardians prior to inclusion into the study.

### 2.2. Subjects

The study subjects consisted of 105 untreated AIS girls aged between 9 and 17 years and 40 age-matched non-AIS girls. The patients were recruited from our clinic and diagnosed by clinical assessments and standard standing roentgenographic examinations from June 1, 2014, to March 15, 2015. The healthy girls were recruited from local schools and examined by physical examination and X-ray examination to eliminate any spinal deformity. All AIS girls need to meet the following inclusion criteria: the minimum of Cobb angle of 10°; follow-up clinical assessments, which were conducted at an interval of 3 to 6 months until 18 months; complete records of pubertal growth, including age, Risser grade, menstrual status, and anthropometric measurements. And the exclusion criteria for AIS and control girls were congenital vertebral malformations, neuromuscular diseases, metabolic diseases, skeletal dysplasia, connective tissue abnormalities, mental retardation, a history of recent steroid intake, or other diseases affecting bone metabolism and BMIs >23.0 (according to the World Health Organization standard for overweight criteria in Asian populations).

This was a prospective observational study. The patients were followed clinically every three to six months. Curve severity was monitored by standard scoliosis radiographs examinations. The criteria for brace treatment were established in accordance with 2005 brace studies criteria of Scoliosis Research Society (SRS). After 18-month follow-up, the patients were divided into 2 groups: progression group whose main Cobb angle exceeded 40° or increased by 6° despite bracing and/or undergoing surgery; stable group whose main Cobb angle increased by less than 5° during the clinical follow-ups.

### 2.3. Anthropometric and Radiographic Assessments

The physical characteristics were recorded at the time of diagnosis. Standing height was measured to the nearest 0.1 cm. Weight (in underwear) was measured to 0.1 kg. The corrected height was computed by using Bjure's formula (log⁡Y = 0.011X − 0.177), where Y is the loss of trunk height (cm) due to the spinal deformity and X is the main curve Cobb angle. Body mass index (BMI) was calculated by dividing weight (kg) with height squared (m2) and corrected BMI (cBMI) by dividing weight (kg) with corrected height squared (m2). The Risser grade and curve magnitude were obtained from a full-length standing posteroanterior radiograph of the total spine.

### 2.4. Measurements of Ghrelin and Leptin

Peripheral venous blood samples were collected from all subjects in the morning after an overnight fasting without a large load of sports from all subjects in the follicular phase, and the serum samples were separated after centrifugation and stored at −80°C until assay. Total ghrelin and leptin levels were measured by using enzyme-linked immunosorbent assay (ELISA) kits (Elabscience Biotechnology, Wuhan, Hubei, China) with minimum detectable values of 0.09 ng/ml and 48.88 pg/ml, respectively.

### 2.5. Statistical Analysis

Data are presented as mean ± SD. The comparison of anthropometric data between AIS and control groups was computed using Mann–Whitney *U* test. Serum ghrelin and leptin levels were compared between AIS and control groups using independent samples *t*-test. The statistical significance was set at P < 0.05. Pearson or Spearman's rank correlations between ghrelin and leptin levels and growth-related parameters (age, weight, corrected height, corrected BMI, main Cobb angle, and Risser sign) were analyzed in AIS group. As the physical characteristics and circulating leptin and ghrelin levels were continuous variables, appropriate transformations on continuous variables into categorical variables were performed. Specifically, age, cBMI, main Cobb angle, and Risser sign of AIS girl were classified into 2 grades (high level and low level) by median. In detail, the age was subdivided into 9 to 12 years (code 0) and 13 to 17 years (code 1), the cBMI was subdivided into less than 16.8 kg/m^2^ (code 0) and more than or equal to 16.8 kg/m^2^ (code 1), the main Cobb angle was subdivided into 10° to 22° (code 0) and more than or equal to 23° (code 1), and the Risser sign was subdivided into 0-2 (code 0) and 3-5 (code 1). The ghrelin and leptin levels were classified into 3 grades (high, middle, and low level) by tertiles: the ghrelin level was categorized as ≤5.00 ng/ml (code 0), 5.00-7.30 ng/ml (code 1), and ≥7.30 ng/ml (code 2); the leptin levels were categorized as ≤5.13 ng/ml (code 0), 5.13-7.44 ng/ml (code 1), and ≥7.44 ng/ml (code 2). The *χ*2 test was carried out to compare the differences of the distributions of the parameters between curve progression and stable curve AIS girls. The univariate analysis was used to assess the association between curve progression and each independent variable. The variables with P value < 0.2 were included in the regression model. Multivariate logistic regression with stepwise elimination was utilized to identify the prognostic factor(s) for curve progression. Odds ratios (ORs) were determined from the multivariate analysis. Receiver operating characteristic (ROC) curves for the accuracy of ghrelin and leptin in predicting the curve progression were carried out, and analysis was performed to determine the optimal cut-off points from the curves.

## 3. Results

**Figure 1:**
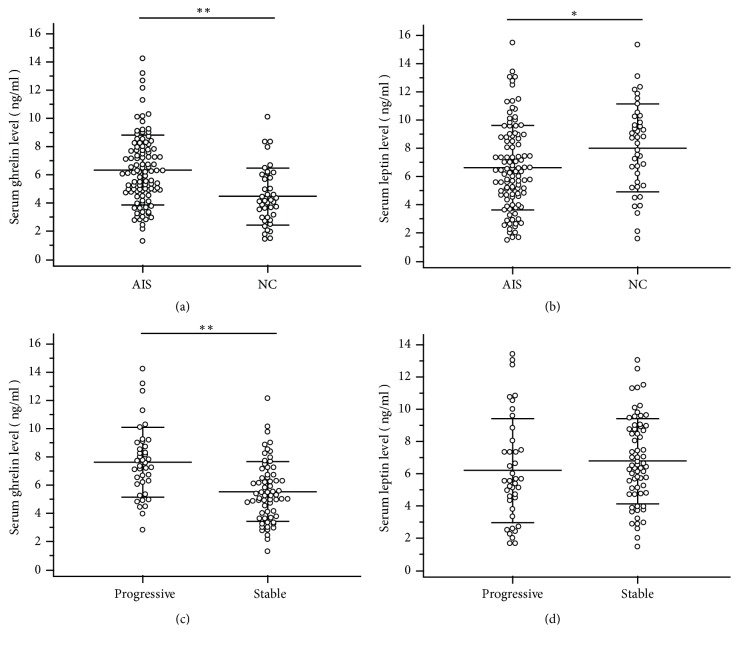
Serum levels of ghrelin and leptin were measured in AIS girls and control group using ELISA kit (*∗ P* < 0.05, *∗∗ P* < 0.01). (a, b) Comparison between AIS girls and control girls. (c, d) Comparison between curve progression and curve stability AIS girls. AIS, adolescent idiopathic scoliosis; NC, negative controls.

A total of 105 AIS girls were enrolled over the above-mentioned period, and 40 age-matched non-AIS girls were included in the study. During the follow-up, there were 40 patients (38.10% of all AIS patients) whose main Cobb angle exceeded 40° or increased by 6°. Thus, the AIS patients can be categorized into two groups: progression and stable. The physical characteristics of the patients in progression and stable group and control group are shown in Table 1. It was found that the mean age, weight, height, and corrected height between AIS group and control group had no significant difference. However, AIS patients showed lower BMI and cBMI than controls (17.55 ± 1.35/16.89 ± 1.31 vs. 18.17 ± 1.36 kg/m^2^,* p* < 0.01). For AIS patients with curve progression, significantly lower BMI and cBMI (17.09 ± 1.49 vs. 17.83 ± 1.18 kg/m^2^,* p* < 0.01; 16.35 ± 1.35 vs. 17.22 ± 1.17 kg/m^2^,* p* < 0.01) and higher main curve Cobb angle (28.88 ± 13.80 vs. 21.55 ± 6.39°,* p* < 0.01) were found, compared with those with stable curves. Normality test showed that serum total ghrelin and leptin levels are normally distributed in both AIS and control groups (results not shown). As shown in Figure 1, a significantly lower leptin level (6.55 ± 2.88 vs. 8.01 ± 3.12 ng/ml,* p* < 0.05) and higher ghrelin level (6.33 ± 2.46 vs. 4.46 ± 2.02 ng/ml,* p* < 0.05) were found in all AIS patients. Curve progression patients had a higher ghrelin level than stable curve patients (7.61 ± 2.48 vs. 5.54 ± 2.11 ng/ml,* p* < 0.01); for leptin level, there was no significant difference between progression and stable group.

The distribution of physical characteristics and biochemical markers in each group was displayed in Table 2. Apparently, the rate of curve progression was significantly higher in younger (age < 12 years) than in older patients (65.0% vs. 35.0%,* p* = 0.048), in patients with lower cBMI (cBMI < 16.8 kg/m^2^) than those with higher cBMI (67.5% vs. 32.5%,* p* < 0.05), in patients with larger initial curve magnitude (main Cobb angle ≥ 23°) than those with smaller initial curve magnitude (65.0% vs. 35.0%,* p* < 0.01), in premenarche patients than postmenarche ones (67.5% vs. 32.5%,* p* < 0.05), and in patients with high ghrelin level (ghrelin ≥ 7.30 ng/ml) than those with low ghrelin level (52.5% vs. 47.5%,* p* < 0.01). In addition, the rate of curve progression had no significant difference in terms of Risser sign or leptin level.  Table 3 showed the relationships between serum ghrelin and leptin level of AIS girls and growth-related parameters (age, corrected height, weight, corrected BMI, initial Cobb angle, menstrual status, and Risser sign). Apparently, ghrelin level was significantly negatively correlated with Risser sign (r = −0.489; p < 0.01), menstrual status (r = −0.239; p < 0.001), corrected BMI (r = −0.208; p < 0.001), and age (r = −0.303; p < 0.05). In contrast, leptin level was significantly positively correlated with Risser sign (r = 0.378; p < 0.01), weight (r = 0.325; p < 0.01), corrected BMI (r = 0.252; p < 0.05), corrected height (r = 0.264; p < 0.001), and age (r = 0.302; p < 0.05), while initial Cobb angle of AIS girls correlated neither with ghrelin level nor with leptin level.

**Figure 2:**
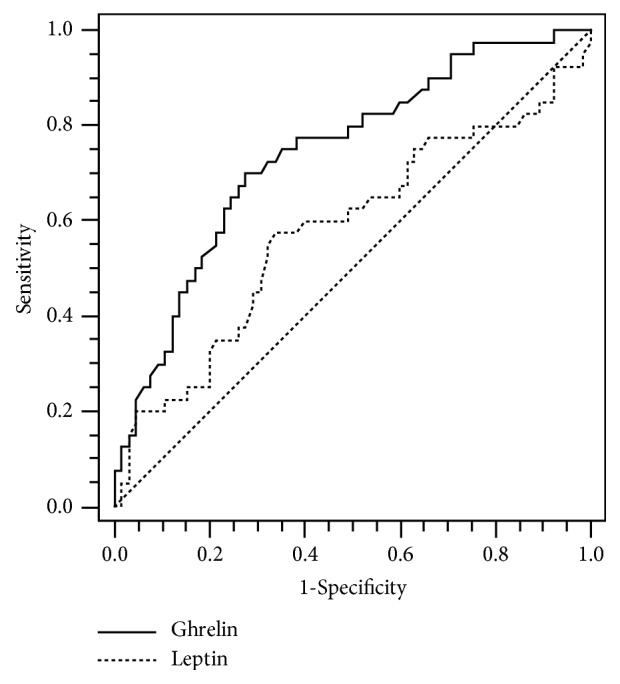
Receiver operator characteristic (ROC) curves for the accuracy of ghrelin and leptin to predict the curve progression in AIS girls were analyzed. Ghrelin levels of >6.48 ng/ml were predictive for curve progression with 70.00 % sensitivity and 72.31 % specificity, and the area under the curve (AUC) was 0.741 (95 % confidence interval 0.646-0.821). Leptin levels of ≤5.67 ng/ml were predictive for curve progression with 57.50 % sensitivity and 66.15 % specificity, and the AUC was 0.580 (95 % confidence interval 0.480–0.676).

The results of multivariate logistic stepwise regression showed that premenarche status, initial main Cobb magnitude that was more than or equal to 23°, high ghrelin level (≥7.30 ng/ml), and lower Risser grade (grades 0 to 2) were identified as risk factors in predicting curve progression (Table 4). The predictive power of these prognostic factors was indicated by odds ratio (OR) within the 95% confidence interval (95% CI). The OR of the initial menstrual status (*P* = 0.003) stands at 0.174 (95% CI, 0.055-0.553); the OR of the initial Cobb angle (*P* = 0.067) stands at 2.391 (95% CI, 0.939-6.084); the OR of the ghrelin level (*P* < 0.001) stands at 3.475 (95% CI, 1.709-7.064); and the OR of the initial Risser grade (*P* = 0.020) stands at 4.877 (95% CI, 1.282-18.545). As shown in Figure 2, the receiver operating characteristic (ROC) analysis showed that the optimal cut-off of ghrelin values was 6.48 ng/ml with the area under the curve that was 0.741 (95% confidence interval 0.646 to 0.821), and at this point, the sensitivity and the specificity for curve progression were 70.00 % and 72.31 %, respectively; the optimal cut-off values of leptin were 5.67 ng/ml with the area under the curve that was 0.580 (95% confidence interval 0.480 to 0.676), and at this point the sensitivity and the specificity for curve progression were 57.50 % and 66.15 %, respectively.

## 4. Discussion

AIS is the most prevalent spinal deformity disease with a complex etiology. Until now, we still do not have a reliable method to predict the level of curve severity at the first session. Previous studies have reported various characteristics and parameters with their ability to predict curve progression in AIS patients. The results of these studies showed that the risk of curve progression correlated with growth potential and curve magnitude [21]. Several hormones have been identified as influential factors in individual's growth and development [22–25]. More precisely, leptin and ghrelin are two key factors among them, which have been shown to influence physical growth and glucose/lipid metabolism [26]. Leptin, a 16 kDa protein secreted by adipocytes, is an important factor affecting skeletal growth. Ghrelin is secreted from the stomach and plays a role in regulation of meal initiation and insulin secretion. It has been suggested that abnormal growth pattern and lower BMI may correlate with leptin and ghrelin level disorder in AIS girls. Several studies have reported that circulating level of leptin is low and the circulating level of ghrelin is high in AIS girls. In the study reported by Qiu et al. [17] a low circulating leptin level was reported in AIS patients. Sales de Gauzy et al. [18] have reported that serum ghrelin level was 1.8-fold higher in AIS girls than in control group. Meanwhile, leptin level was found to be correlated with growth potential parameters, and the correlation between ghrelin and age was also found in AIS girls. In the current study, low concentration of serum leptin and high concentration of serum ghrelin were observed in the AIS group, which was in accordance with previous studies. More importantly, according to the analysis of the correlations between physical characteristics and leptin/ghrelin levels, leptin level significantly correlated with age, Risser sign, weight, and other growth parameters, and ghrelin level negatively correlated with Risser sign, age, and corrected BMI, indicating that serum leptin and ghrelin levels may be risk factors in curve progression AIS girls.

Several previous studies have reported that different characteristics can be used for the prediction of severe spine deformity, such as clinical [27], radiographic [28], physiologic [29], biochemical [30], genetic [31], and combinatorial parameters [32]. Tan et al. [21] examined 186 AIS patients and noted that curve magnitude more than 30 degrees at first session was the most important predictive factor for curve progression. A prospective study performed by Hung et al. [33] showed that osteopenia, lower Risser grade, and larger initial Cobb angle correlated with curve progression. A DNA-based prognostic test named “ScoliScore” has been developed by Ward K et al. [11] provided important information in risk prediction, but the test applies only to white AIS patients. In addition, Noshchenko A et al. [10] analyzed the reported predictive values of different factors in progressive AIS patients in a systematic review; the result showed that current risk factors are not recommended for clinical use as diagnostic criteria due to low level of evidence. Overall, more prospective cohort studies and randomized controlled clinical trials (RCTs) were needed to develop more functional approaches in the prediction of curve progression in scoliosis.

The prognostic factors that were identified in the study were menstrual status, Risser sign, ghrelin level, and initial Cobb angle. The ghrelin level in progressive group was significantly higher than in stable curve group and may predict the curve progression of AIS girls. However, menstrual status, Risser sign, and Cobb magnitude have been found to be important risk factors in previous studies. The mechanism for the increase of ghrelin concentration in progressive AIS girls was explored. In the general population, an association between circulating ghrelin level disorder and delayed puberty was observed [34]. Therefore, high ghrelin level may contribute to puberty delay in AIS patients. Furthermore, ghrelin regulates bone remodeling in vivo and increases proliferation and differentiation of osteoblasts in vitro [35]. Ghrelin increases osteoblast differentiation and proliferation by modulating the CREB and Runx2 pathways [35]. Leptin also increases osteoblast proliferation and activity [36–38]. Clinically, low bone mineral density or osteopenia was widely identified in AIS girls [39]. Thus, it is likely that ghrelin dysregulation may be the consequence of scoliosis as a feedback regulation, but without being a causal factor. However, it is known that ghrelin level is influenced by various factors, such as metabolic status, other hormone levels, and ghrelin resistance. It interacts with leptin in modulating bone structure [40]; their combined effect in bone metabolism and energy metabolism still needs further investigation.

There are several limitations in this study. First, all the patients were followed for 18 months, so parts of them did not have skeletal maturity in the end of follow-up. Thus, the results of the study may not be able to represent the general trends. Second, hormone levels were not monitored continuously, which resulted in the loss of fluctuation pattern of ghrelin and leptin levels in different kinds of patients. Further studies on the dynamic changes of those hormones in AIS girls were needed. Third, the patients under brace treatment were also included, which may have effects on curve progression. Hence, it would be ideal to set a separate group for the patients under brace treatment.

In conclusion, the findings indicated marked increase in serum ghrelin levels and decrease in serum leptin levels in AIS girls. The correlations between the two hormones and growth parameters were documented. Moreover, high ghrelin level may serve as a new quantitative indicator for predicting curve progression and thus helps in precise selection of appropriate treatment in AIS girls. To our knowledge, this is the first cohort study demonstrating the close correlation between serum ghrelin levels and the classic curve progression predictors in AIS patients. The clinical importance of the present study is that serum ghrelin could be a risk factor, linked with other reported prognostic factors, to predict the final outcome of scoliosis. Therefore, further large sample cohort studies are needed in this field.

## Figures and Tables

**Table 1:** Physical characteristics and circulating leptin and ghrelin levels between AIS and controls group.

Items	AIS group			Controls (n = 40)
	Progressive Group (n = 40)	Stable Group (n = 65)	Total (n = 105)	
Age (yr)	12.08 ± 1.56	12.65 ± 2.06	12.43 ± 1.90*∗*	12.80 ± 1.22
Weight (kg)	39.10 ± 5.75	41.42 ± 6.49	40.54 ± 6.29	42.78 ± 5.67
Height (cm)	150.90 ± 7.49	151.99 ± 9.69	151.57 ± 8.90	153.12 ± 5.86
cHeight (cm)	154.29 ± 8.29	154.61 ± 9.69	154.49 ± 9.14	-
BMI (kg/m^2^)	17.09 ± 1.49‡	17.83 ± 1.18	17.55 ± 1.35*∗∗*	18.17 ± 1.36
cBMI (kg/m^2^)	16.35 ± 1.35‡	17.22 ± 1.17	16.89 ± 1.31*∗∗*	-
Main curve Cobb angle (°)	28.88 ± 13.80‡	21.55 ± 6.39	24.34 ± 10.45	-
Risser sign	1.83 ± 1.52	2.17 ± 1.84	2.04 ± 1.76	2.35 ± 1.63
Years since menarche (yr)	0.82 ± 0.45	0.91 ± 0.60	0.89 ± 0.57	0.86 ± 0.55
Leptin (ng/ml)	6.19 ± 3.22	6.77 ± 2.66	6.55 ± 2.88*∗*	8.01 ± 3.12
Ghrelin (ng/ml)	7.61 ± 2.48‡	5.54 ± 2.11	6.33 ± 2.46*∗∗*	4.46 ± 2.02

Values are shown as means ± SD. In controls, corrected height (cHeight) and corrected BMI (cBMI) were equal to height, BMI, of them, respectively. AIS indicates adolescent idiopathic scoliosis; BMI, body mass index. †  *P* < 0.05 and ‡  *P* < 0.01 between progressive AIS group and stable AIS group; *∗*  *P* < 0.05 and *∗∗*  *P* < 0.01 between AIS girls and control girls.

**Table 2:** Comparisons of distribution of physical characteristics and circulating leptin and ghrelin levels between progressive AIS group and stable AIS group.

Items	Progressive Group (n = 40)	Stable Group (n = 65)	*P *value
Age			
<12 years	26 (65.0%)	28 (43.1%)	0.048
≥12 years	14 (35.0%)	37 (56.9%)	
cBMI			
<16.8 kg/m^2^	27 (67.5%)	26 (40.0%)	0.011
≥16.8 kg/m^2^	13 (32.5%)	39 (60.0%)	
Main cobb angle			
<23°	14 (35.0%)	41 (63.1%)	0.009
≥23°	26 (65.0%)	24 (36.9%)	
Menstrual status			
Pre-menarche	27 (67.5%)	26 (40.0%)	0.011
Post-menarche	13 (32.5%)	39 (60.0%)	
Ghrelin			
≤5.00 ng/ml	7 (17.5%)	29 (44.6%)	<0.001
5.00-7.30 ng/ml	12 (30.0%)	24 (36.9%)	
≥7.30 ng/ml	21 (52.5%)	12 (18.5%)	
Leptin			
≤5.13 ng/ml	17 (42.5%)	19 (29.2%)	0.360
5.13-7.44 ng/ml	12 (30.0%)	22 (33.8%)	
≥7.44 ng/ml	11 (27.5%)	24 (36.9%)	
Risser sign			
0-2	26 (65.0%)	40 (61.5%)	0.882
3-5	14 (35.0%)	25 (38.5%)	

Values are shown as no. (%). BMI, body mass index.

**Table 3:** The correlations between circulating ghrelin and leptin levels and physical parameters in AIS girls.

R Value	Age	cHeight	Weight	cBMI	Cobb	Menstrual Status	Risser Sign
Ghrelin	-0.303*∗*	-0.110	-0.183	-0.208‡	0.080	-0.239‡	-0.489†
Leptin	0.302*∗*	0.264‡	0.325†	0.252*∗*	0.027	0.143	0.378†

R represents Pearson's correlation coefficients for normative data and Spearman's correlation coefficients for nonnormative data.

Symbols indicate significant correlation: *∗* P < 0. 05; † P < 0.01; ‡ P < 0.001.

**Table 4:** Multivariate logistic regression analysis of curve progression predictive factors.

Items	Regression coefficient (B)	Odds Ratio (95%CI)*∗*	*P *value
Menstrual status at inclusion	1.748	0.174 (0.055-0.553)	0.003
Cobb angle at inclusion	0.872	2.391 (0.939-6.084)	0.067
Ghrelin level at inclusion	1.245	3.475 (1.709-7.064)	<0.001
Risser sign at inclusion	1.584	4.877 (1.282-18.545)	0.020

95% CI indicates 95% confidence interval of odds ratio; CI, confidence interval.

## Data Availability

The data used to support the findings of this study are included within the article's Supplementary Materials.

## Supplementary Materials

Supplementary Materials The patient characteristics and biochemical data used to support the findings of this study are included within the supplementary information file. AIS group data were displayed in sheet 1; control group data were displayed in sheet 2.

## References

[1] Weinstein S. L., Dolan L. A., Cheng J. C., Danielsson A., Morcuende J. A. (2008). Adolescent idiopathic scoliosis. *The Lancet*.

[2] Asher M. A., Burton D. C. (2006). Adolescent idiopathic scoliosis: Natural history and long term treatment effects. *Scoliosis*.

[3] Zhang H., Guo C., Tang M. (2015). Prevalence of scoliosis among primary and middle school students in mainland china: A systematic review and meta-analysis. *The Spine Journal*.

[4] Liu L., Xiu P., Li Q., Song Y., Chen R., Zhou C. (2010). Prevalence of cardiac dysfunction and abnormalities in patients with adolescent idiopathic scoliosis requiring surgery. *Orthopedics*.

[5] Hengwei F., Zifang H., Qifei W. (2016). Prevalence of idiopathic scoliosis in Chinese schoolchildren. *The Spine Journal*.

[6] Busscher I., Wapstra F. H., Veldhuizen A. G. (2010). Predicting growth and curve progression in the individual patient with adolescent idiopathic scoliosis: Design of a prospective longitudinal cohort study. *BMC Musculoskeletal Disorders*.

[7] Ylikoski M. (2005). Growth and progression of adolescent idiopathic scoliosis in girls. *Journal of Pediatric Orthopaedics B*.

[8] Zhang H., Zhao S., Zhao Z. (2014). The association of rs1149048 polymorphism in Matrilin-1(MATN1) gene with adolescent idiopathic scoliosis susceptibility: A meta-analysis. *Molecular Biology Reports*.

[9] Agabegi S. S., Kazemi N., Sturm P. F., Mehlman C. T. (2015). Natural History of Adolescent Idiopathic Scoliosis in Skeletally Mature Patients: A Critical Review. *Journal of the American Academy of OrthopaedicSurgeons *.

[10] Noshchenko A., Hoffecker L., Lindley E. M. (2015). Predictors of spine deformity progression in adolescent idiopathic scoliosis: A systematic review with meta-analysis. *World Journal of Orthopedics*.

[11] Ward K., Ogilvie J. W., Singleton M. V., Chettier R., Engler G., Nelson L. M. (2010). Validation of DNA-based prognostic testing to predict spinal curve progression in adolescent idiopathic scoliosis. *The Spine Journal*.

[12] Chen X. X., Yang T. (2015). Roles of leptin in bone metabolism and bone diseases. *Journal of Bone and Mineral Metabolism*.

[13] Delporte Christine (2013). Structure and Physiological Actions of Ghrelin. *Scientifica*.

[14] Wee N. K., Baldock P. A. (2014). The hunger games of skeletal metabolism. *BoneKEy Reports*.

[15] Liang G., Gao W., Liang A. (2012). Normal leptin expression, lower adipogenic ability, decreased leptin receptor and hyposensitivity to leptin in adolescent idiopathic scoliosis. *PLoS ONE*.

[16] Liu Z., Tam E. M. S., Sun G.-Q. (2012). Abnormal leptin bioavailability in adolescent idiopathic scoliosis: an important new finding. *The Spine Journal*.

[17] Qiu Y., Sun X., Qiu X. (2007). Decreased circulating leptin level and its association with body and bone mass in girls with adolescent idiopathic scoliosis. *The Spine Journal*.

[18] Sales de Gauzy J., Gennero I., Delrous O., Salles J.-P., Lepage B., Accadbled F. (2015). Fasting total ghrelin levels are increased in patients with adolescent idiopathic scoliosis. *Scoliosis*.

[19] Tam E. M. S., Yu F. W. P., Hung V. W. Y. (2014). Are volumetric bone mineral density and bone micro-architecture associated with leptin and soluble leptin receptor levels in adolescent idiopathic scoliosis? - A case-control study. *PLoS ONE*.

[20] Wang Y.-J., Yu H.-G., Zhou Z.-H., Guo Q., Wang L.-J., Zhang H.-Q. (2016). Leptin receptor metabolism disorder in primary chondrocytes from adolescent idiopathic scoliosis girls. *International Journal of Molecular Sciences*.

[21] Tan K.-J., Moe M. M., Vaithinathan R., Wong H.-K. (2009). Curve progression in idiopathic scoliosis: Follow-up study to skeletal maturity. *The Spine Journal*.

[22] Lowe T., Lawellin D., Smith D. (2002). Platelet calmodulin levels in adolescent idiopathic scoliosis: Do the levels correlate with curve progression and severity?. *The Spine Journal*.

[23] Kindsfater K., Lowe T., Lawellin D., Weinstein D., Akmakjian J. (1994). Levels of platelet calmodulin for the prediction of progression and severity of adolescent idiopathic scoliosis. *The Journal of Bone & Joint Surgery*.

[24] Brodner W., Krepler P., Nicolakis M. (2000). Melatonin and adolescent idiopathic scoliosis. *The Journal of Bone & Joint Surgery (British Volume)*.

[25] Kulis A., Goździalska A., Drąg J. (2015). Participation of sex hormones in multifactorial pathogenesis of adolescent idiopathic scoliosis. *International Orthopaedics*.

[26] Schorr M., Miller K. K. (2017). The endocrine manifestations of anorexia nervosa: Mechanisms and management. *Nature Reviews Endocrinology*.

[27] Sun X., Wang B., Qiu Y. (2010). Outcomes and predictors of brace treatment for girls with adolescent idiopathic scoliosis. *Orthopaedic Surgery*.

[28] Modi H. N., Suh S. W., Song H.-R., Yang J.-H., Ting C., Hazra S. (2009). Drooping of apical convex rib-vertebral angle in adolescent idiopathic scoliosis of more than 40 degrees: A prognostic factor for progression. *Journal of Spinal Disorders & Techniques*.

[29] Lam T. P., Hung V. W. Y., Yeung H. Y. (2013). Quantitative Ultrasound for Predicting Curve Progression in Adolescent Idiopathic Scoliosis: A Prospective Cohort Study of 294 Cases Followed-Up Beyond Skeletal Maturity. *Ultrasound in Medicine & Biology*.

[30] Akoume M.-Y., Franco A., Moreau A. (2013). Cell-based assay protocol for the prognostic prediction of idiopathic scoliosis using cellular dielectric spectroscopy.. *Journal of visualized experiments : JoVE*.

[31] Bohl D. D., Telles C. J., Ruiz F. K., Badrinath R., DeLuca P. A., Grauer J. N. (2016). A Genetic Test Predicts Providence Brace Success for Adolescent Idiopathic Scoliosis When Failure Is Defined as Progression to >45 Degrees. *Clinical Spine Surgery*.

[32] Ogura Y., Takahashi Y., Kou I. (2013). A replication study for association of 53 single nucleotide polymorphisms in a scoliosis prognostic test with progression of adolescent idiopathic scoliosis in Japanese. *The Spine Journal*.

[33] Hung V. W. Y., Qin L., Cheung C. S. K. (2005). Osteopenia: a new prognostic factor of curve progression in adolescent idiopathic scoliosis. *The Journal of Bone & Joint Surgery*.

[34] Cheng Y., Hong M., Cheng B. (2014). Impact of changes in postnatal nutrition on puberty onset and the expression of hypothalamic GnRH and ghrelin. *European Review for Medical and Pharmacological Sciences*.

[35] Ma C., Fukuda T., Ochi H. (2015). Genetic determination of the cellular basis of the ghrelin-dependent bone remodeling. *Molecular Metabolism*.

[36] Turner R. T., Kalra S. P., Wong C. P. (2013). Peripheral leptin regulates bone formation. *Journal of Bone and Mineral Research*.

[37] Wu N., Wang Q., Li H., Wu X., Sun Z., Luo X. (2010). Corrigendum to “Relationships between serum adiponectin, leptin concentrations and bone mineral density and bone biochemical markers in Chinese women” [Clinica Chimica Acta 411 (2010) 771–775]. *Clinica Chimica Acta*.

[38] Zhang H., Xie H., Zhao Q. (2010). Relationships between serum adiponectin, apelin, leptin, resistin, visfatin levels and bone mineral density, and bone biochemical markers in post-menopausal Chinese women. *Journal of Endocrinological Investigation*.

[39] Cheng J. C., Tang S. P., Guo X., Chan C. W., Qin L. (2001). Osteopenia in adolescent idiopathic scoliosis: a histomorphometric study. *The Spine Journal*.

[40] Delhanty P. J. D., van der Eerden B. C. J., van Leeuwen J. P. T. M. (2014). Ghrelin and bone. *BioFactors*.

